# Our Advertising Department

**Published:** 1878-08-20

**Authors:** 


### OUR ADVERTISING DEPARTMENT.

Our advertising department is growing, and is becoming an important feature of our journal. A subscriber has complained of this, supposing that we drew upon our space for reading matter to make room for advertisements. This is a mistake. Our reading matter is never diminished, but remains the same, and the advertising is thrown in, so far as our readers are concerned. The more advertisements we have the better able are we to supply a good journal.

### TILDEN & CO.

See advertisement by the above large and well known house of their Ergot and Bromo-chloralum.

### LOEFLUND'S EXTRACT OF MALT AND LIEBIG'S FOOD FOR INFANTS.

We ask special attention to the advertisement of the above article by Albert C. Dung, Druggist, New York.

### FRANKLIN PRINTING HOUSE.

We invite attention to the advertisement of this large and popular printing establishment in Atlanta.

### REED & CARNRICK.

Notice carefully the two page advertisement of the above large and well established house in the present issue of our journal.

